# Supplementary material for: Djnedd4L Is Required for Head Regeneration by Regulating Stem Cell Maintenance in Planarians
Source: Int J Mol Sci. 2021 Oct 28;22(21):11707. doi: 10.3390/ijms222111707 (PMC8583885; doi:10.3390/ijms222111707)
Supplement: Supplementary file 1 [file ijms-22-11707-s001.zip › Supplementary Material/Supplementary Figures and Table.pdf]

Supplementary Information

SUPPLEMENTARY INFORMATION INDEX

- Supplementary Figures S1, S2

- Supplementary Table S1

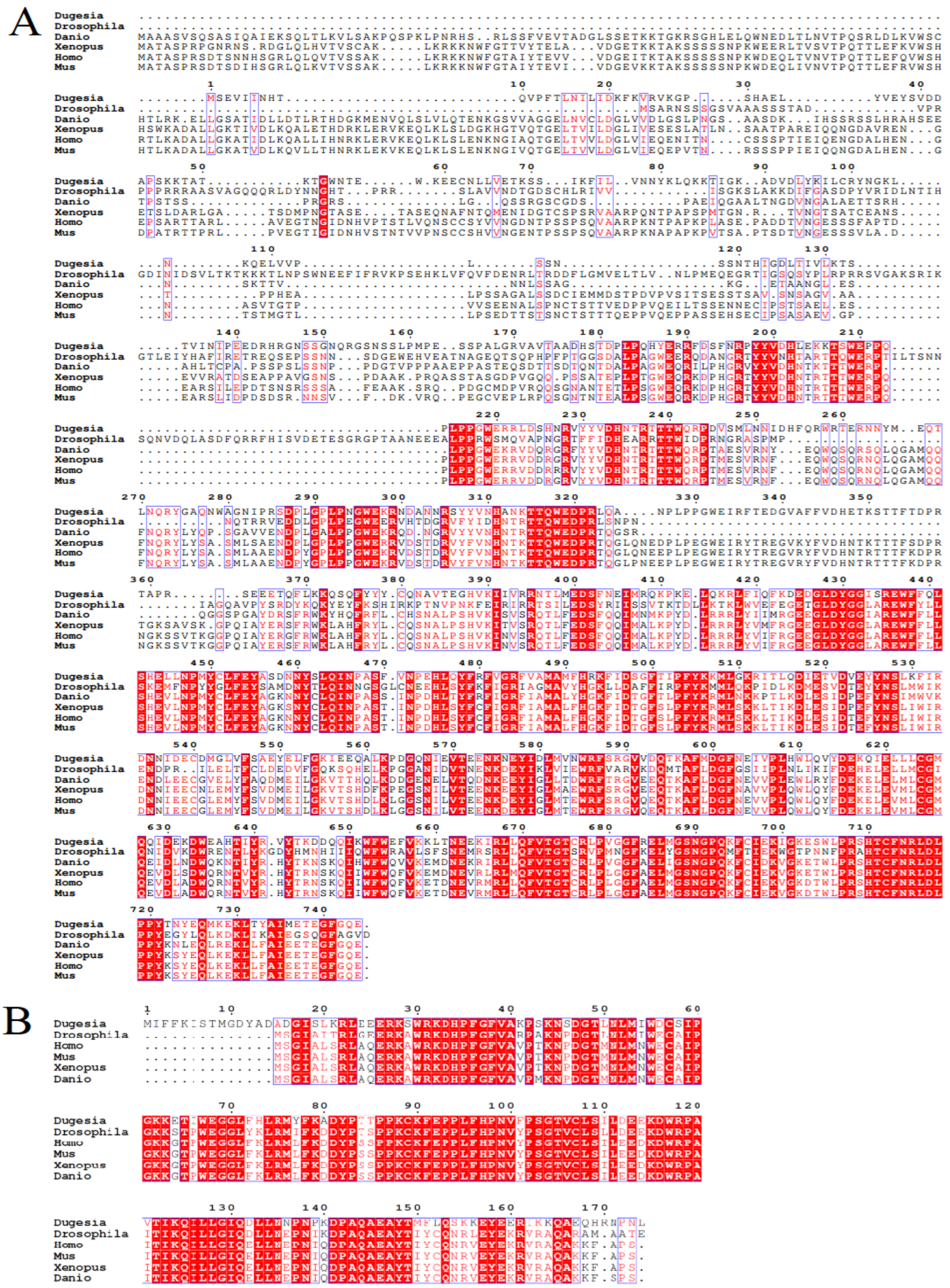

Figure 1. Multiple sequence alignment of Djubc9 and Djneddl4 from *Dugesia japonica*, *Homo sapiens*, *Mus musculus*, *Drosophila serrata*, *Xenopus laevis*, and *Danio rerio*. (**A**) Djneddl4. (**B**) Djubc9. Absolutely conserved residues are in red shaded boxes, and highly conserved residues are colored red and are boxed.

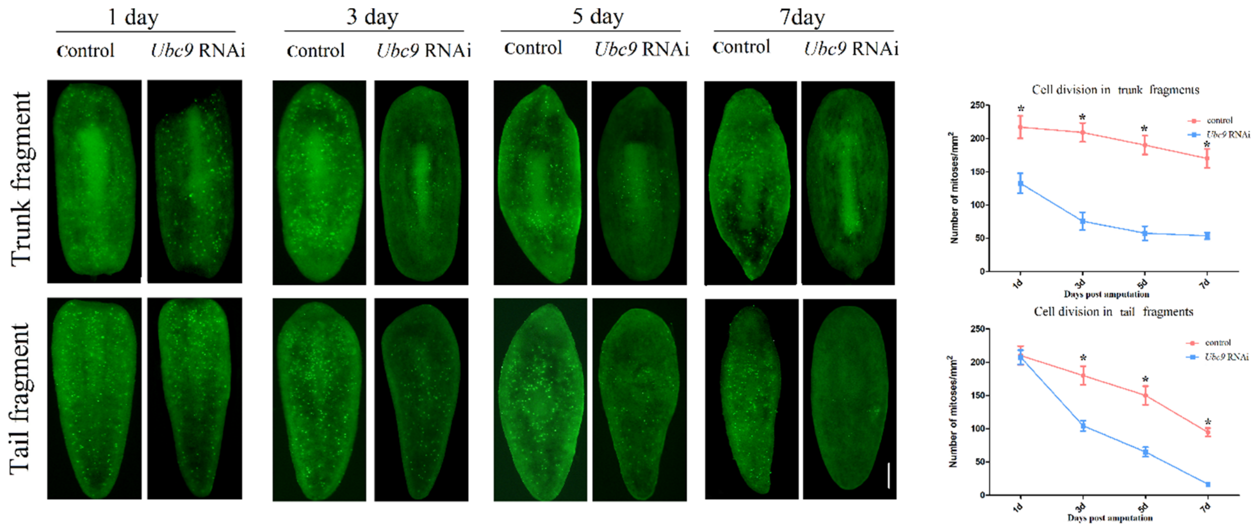

Figure 2. Analysis of the effect of *Djubc9* RNAi on cell division in regenerating fragments. Cell division decreased in the *Djubc9 (RNAi)* phenotype in regenerating fragments. Statistical differences are measured by Student's *t*-test and error bars indicate s.e.m. For each time point, n=15 with three experimental replicates. Scale bars: 200 μm.

Table S1. PCR primers used in this study.

Djubc9F	CAACTATGGGTGATTATGCAGATG
Dj ubc9R	CATGACGACCACGCAAATATATTAC
T7-Djubc9F	TAATACGACTCACTATAGG CATGACGACCACGCAAATATATTAC
T7-Djubc9R	TAATACGACTCACTATAGG CATGACGACCACGCAAATATATTAC
Djneddl4F	CCCAGAACTGCACCAAGAAGTGC
Dj neddl4R	CCAGCCCCATATCGCATTCATC
T7-Djneddl4F	TAATACGACTCACTATAGG CCCAGAACTGCACCAAGAAGTGC
T7-Djneddl4F	TAATACGACTCACTATAGG CCAGCCCCATATCGCATTCATC
qPCR-Dj neddl4F	CCAGAACTGCACCAAGAAGTGC
qPCR-Dj neddl4R	GGAGATCTTTGGGCTTCTGTC
qPCR-Djrfc2F	TGGTATTGATGTGGTGCGTAAT
qPCR-Djrfc2R	CAGTCATACTGTCGCCTTCATC
qPCR-Dj chat F	GTAGCGCCAGTAGTGTCAATAG
qPCR-Dj chat R	CCATAAATCGGCAACCCAATTC
qPCR-Dj nutum F	CGTCGTCTGGAGTCGTTAATC
qPCR-Dj nutum R	GCGTCAGCACATCGAGATAA
qPCR-Dj otxa F	GGCAGTTGTTATGTAGCTGGA
qPCR-Dj otxa R	CCAACTGGAGAACCGATCATATT
qPCR-Dj sfrp-1 F	GGCAATCGTTCCAAAGCGG
qPCR-Dj sfrp-1 R	CCATCGGGTAATTTCGATTTGC

qPCR-Dj axinB F	CCAGTATCGAGTTGTTCGAGTT
qPCR-Dj axinB R	CGGTGGGTCGATTTCTGATAG
qPCR-Dj abd-Ba F	GCTACCTTCGTCTGCATCAA
qPCR-Dj abd-Ba R	GAAGACGTCGCATCTATCCAATA
qPCR-Dj fz-T F	GAACCGAAGAAATAGACC
qPCR-Dj fz-T R	CTACAAATGCCTTGGCCGG
qPCR-Dj wntp1 F	GGGAACTACAGGACGAGTTTG
qPCR-Dj wntp1 R	CAACTACGCAACACCAAATGAA
